# Parental Education Predicts Longitudinal IQ Trajectories in 22q11.2 Deletion Syndrome: A Three‐Cohort European Study

**DOI:** 10.1111/jir.70025

**Published:** 2025-08-04

**Authors:** Yelyzaveta Snihirova, Therese van Amelsvoort, Ann Swillen, Marianne van den Bree, Dennis van der Meer, Janet Harwood, Claudia Vingerhoets, David E. J. Linden

**Affiliations:** ^1^ Mental Health and Neuroscience Research Institute, Department of Psychiatry and Neuropsychology, Faculty of Health, Medicine and Life Sciences Maastricht University Maastricht the Netherlands; ^2^ Centre for Human Genetics University Hospital Gasthuisberg Leuven Belgium; ^3^ Department of Human Genetics KU Leuven Leuven Belgium; ^4^ Centre for Neuropsychiatric Genetics and Genomics Division of Psychological Medicine and Clinical Neurosciences Cardiff Cardiff UK; ^5^ Centre for Precision Psychiatry, Division of Mental Health and Addiction Oslo University Hospital Oslo Norway; ^6^ Institute of Clinical Medicine University of Oslo Oslo Norway; ^7^ Advisium 's Heeren Loo Zorggroep Amersfoort the Netherlands

**Keywords:** 22q11.2 deletion syndrome, cognition, environmental factors, IQ, parental education

## Abstract

**Background:**

22q11.2 deletion syndrome (22q11DS) is a genetic disorder characterised by a wide range of physical, cognitive, and psychiatric symptoms. Current knowledge on 22q11DS highlights considerable variation in cognitive outcomes, but the role of environmental factors in shaping these trajectories over time remains poorly understood.

**Aims:**

This study investigates how environmental factors contribute to variability in intelligence quotient (IQ) among individuals with 22q11DS across three European cohorts. By examining these influences over time, the research aims to identify potential drivers of IQ differences and uncover modifiable factors that may support improved cognitive outcomes in individuals with 22q11DS.

**Methods:**

Data were collected from 297 individuals with 22q11DS across three European cohorts. Cognitive assessments included full‐scale IQ (FSIQ), verbal IQ (VIQ) and performance IQ (PIQ). Environmental measures encompassed parental education, sleep, stress and substance use, gathered through questionnaires and interviews. Baseline associations between environmental measures and IQ were evaluated with ANOVA at the first assessment. To examine within‐person IQ change across three visits, we used linear mixed‐effects models.

**Results:**

We found a significant decline in FSIQ, VIQ and PIQ over time, with linear trends observed for all three measures. Parental education, particularly the father's education, explained a significant proportion of the variance of all IQ‐based measures.

**Conclusions:**

Parental education emerged as a key predictor of IQ, suggesting that socioeconomic factors contribute to cognitive performance variability in individuals with 22q11DS. Even in high‐penetrance genetic variants, such as the 22q11.2 deletion, environmental factors and gene–environment interactions may make significant contributions to the severity of phenotypes.

## 1 Background

22q11.2 deletion syndrome (22q11DS) is a complex genetic disorder caused by the deletion of a segment of chromosome 22 (McDonald‐McGinn et al. 2015; Morrow et al. 2018). The clinical phenotype is highly heterogeneous (Squarcione et al. 2013), often including heart defects, facial anomalies, immunological manifestations, developmental delay (Cirillo et al. 2022) and obesity in adulthood (Voll et al. 2017). Individuals with 22q11DS frequently exhibit social cognitive deficits (Niarchou et al. 2014; Morrison et al. 2020), mental disorders (including anxiety disorder (Schneider et al. 2014), autism spectrum disorder (Schneider et al. 2014; Fiksinski et al. 2017) and psychosis (Murphy et al. 1999; Tang et al. 2014)) and neurocognitive impairments (Morrison et al. 2020), which can manifest as challenges in social interaction (Shashi et al. 2012). The intelligence quotient (IQ) of individuals with 22q11DS is typically lower compared to the general population, with a mean IQ of around 70 (Niarchou et al. 2014), though considerable variability exists (Niarchou et al. 2014). Similar to the general population (Caspi and Moffitt 2006; Tucker‐Drob et al. 2013), variation in cognitive abilities in 22q11DS likely results from additional genetic variation, environmental exposures and their interactions (Olszewski et al. 2014; Snihirova et al. 2022; Fiksinski et al. 2023). Whereas IQ scores are relatively stable over time in the general population (Baxendale 2011), some studies suggest that individuals with 22q11DS experience a notable decline, especially during adolescence (Fiksinski et al. 2021; Vorstman et al. 2015). This decline is often attributed to structural brain changes and the onset of psychiatric comorbidities, such as anxiety and psychosis, which impact cognitive abilities (Chow et al. 2006; Fiksinski et al. 2021). Cognitive impairments in 22q11DS often include deficits in executive function (Campbell et al. 2010), working memory and processing speed (Morrison et al. 2020), which can significantly impact daily functioning and academic performance (Mosheva et al. 2019; Vingerhoets et al. 2024).

Despite the significant role that environmental factors are likely to play, research in 22q11DS has predominantly focused on genetic factors, with relatively less attention given to environmental influences (Snihirova et al. 2022). A limited number of studies have explored the effects of parental factors (Allen et al. 2014; Klaassen et al. 2016), stress (Gur et al. 2021; Palmer et al. 2022) and socioeconomic status (Shashi et al. 2012; Curtin et al. 2022; Pimenta et al. 2024), finding significant associations with the clinical profile of 22q11DS individuals. Yet, the findings from case–control studies, in particular, have been somewhat inconsistent (Shashi et al. 2010; Olszewski et al. 2014; Armando et al. 2018; van Duin et al. 2019; Vingerhoets et al. 2019; Sandini et al. 2020; Schneider et al. 2020; Ilen et al. 2022, 2023; Van de Woestyne et al. 2022; Amir et al. 2023; Modasi et al. 2023; Serur et al. 2023), possibly due to small sample sizes and limited environmental data, resulting in a restricted understanding of the moderating effects of environmental factors in this population.

This explorative study aims to describe the patterns of the environmental influence on three IQ measures (full‐scale IQ, verbal IQ and performance IQ) across three cohorts of 22q11.2 deletion carriers and quantitatively assess the contribution of specific environmental factors to the variability among individuals with 22q11DS. While lower IQ levels in 22q11DS individuals are well‐documented, our study seeks to provide insights into the environmental effects contributing to IQ variability within specific cohorts. We hypothesise that environmental factors significantly influence IQ, contributing to the variability observed within these populations, both cross‐sectionally and longitudinally. The research focuses on parental education, sleep patterns, stress and substance use patterns in individuals with 22q11DS and examines how these factors relate to IQ scores, at both baseline and over time. This study thus aims to identify potentially modifiable environmental factors that could be targeted for interventions to optimise cognitive development in 22q11DS.

## 2 Methods

The authors assert that all procedures contributing to this work comply with the ethical standards of the relevant national and institutional committees on human experimentation and with the Helsinki Declaration of 1975, as revised in 2008. Written informed consent was gained from primary carers and participants. Ethics committees approved the study at the three sites (Cardiff: National Health Service [NHS] Wales Research Ethics Committee; Maastricht: Maastricht University Medical Centre Medical Ethics Committee; Katholieke Universiteit Leuven [KU Leuven]: Research Medical Ethics Committee Universitair Ziekenhuis [UZ] KU Leuven).

### 2.1 Participants

Two hundred ninety‐seven individuals with 22q11DS were recruited from three European sites. Cardiff University recruited children, adolescents and adults with 22q11DS through the Experiences of CHildren with cOpy number variants (ECHO) and Defining Endophenotypes From Integrated NEuroscience (DEFINE) studies (*n* = 93), KU Leuven recruited adolescents and adults with 22q11DS (*n* = 54) and Maastricht University Medical Center+ (MUMC+, Maastricht, the Netherlands) recruited adults with 22q11DS (*n* = 150). Participants were ascertained through similar recruitment methods as described previously (Morrison et al. 2020).

### 2.2 Cognitive Assessments

Full‐scale IQ (FSIQ), verbal IQ (VIQ) and performance IQ (PIQ) were assessed at each site with Wechsler scales in validated local language versions (see Morrison et al. 2020 for more details). All IQ scores were calculated by standardising raw scores for age based on normative data. We used the initial evaluation IQ scores as each participant's base IQ (Timepoint 1) measure. To estimate changes over time, we analysed this base measure and data from the next two consecutive time points across all three cohorts (Timepoints 1, 2 and 3). Not all participants completed all IQ tests (PIQ, VIQ or FSIQ) due to cognitive or behavioural limitations or time constraints, but all completed at least one.

### 2.3 Environmental Measures

#### 2.3.1 Parents' Education

Education information was obtained from questionnaires completed by the participant or primary carer. Parental education level was divided into three groups: high, middle and low. Completion of higher education was considered high level (bachelor's degree and higher) and middle level as completed secondary education (or 12 years of formal education and more). Everything below this level was considered low level according to the International Standard Classification of Education 2011 (ISCED) framework (Schneider 2013).

#### 2.3.2 Sleep

Sleep data were available only for the Cardiff and Maastricht cohorts. Sleep data were collected using different instruments and then harmonised into a single continuous index.

In Cardiff, caregiver interviews employed the Child and Adolescent Psychiatric Assessment (CAPA) sleep section (Angold et al. 1995) to screen for nine symptoms: insomnia, hypersomnia, restless sleep, inadequate rest, nightmares, fatigue, fatigability, somnambulism and night terrors, over the prior 3 months (Moulding et al. 2020) (Table S1). Each symptom was recoded as present (1) if endorsed or absent (0) if not, and these eight binary items were summed to form a raw score for Cardiff.

In Maastricht, self‐report items included sleep onset latency, number and duration of night wakings, wake‐up time, time spent awake on rising and subjective quality (7‐point Likert scale, ranging from −3 to 3, reversed, so higher scores = worse sleep) (Hyde et al. 2021) (Table S2). Each Maastricht variable was standardised (z‐scored), then averaged to yield a raw score for Maastricht.

To enable pooled analyses, both raw scores were converted to site‐specific z‐scores (mean = 0, SD = 1), producing a common ‘Sleep Score’ that reflects relative sleep disturbance within each cohort. Sleep score was used in all subsequent cross‐sectional ANOVAs and longitudinal mixed‐effects models.

#### 2.3.3 Stress

Stressful event data were available only for the Cardiff and Maastricht cohorts.

In Maastricht, participants completed the Dutch 25‐item Childhood Trauma Questionnaire (CTQ) (Bernstein et al. 2003), rated on a 5‐point Likert scale. A general childhood trauma score was generated by summing adverse events.

In Cardiff, a 34‐item Life Events checklist assessed distressing experiences over the past 2 years. Events were coded as present (1) or absent (0), with their impact rated from ‘very unpleasant’ to ‘very pleasant’. Adverse events were coded as ‘1’, and the sum of adverse effects was calculated.

For both cohorts, given the potential impact of a single event, the presence of at least one stressful event was coded as ‘present’ and its absence as ‘absent’.

#### 2.3.4 Substance Use

In Cardiff, substance use was assessed using the CAPA Substance Use section.

In Maastricht and Leuven, cannabis use was measured via sections B, J and L of the Composite International Diagnostic Interview (CIDI) (Wittchen 1994). Participants were dichotomised as users or non‐users based on cannabis use and substance abuse presence. Psychiatric clinical files, based on systematic assessments by psychiatrists, were also reviewed.

Alcohol use was defined as alcohol dependence/abuse (all cohorts) or consuming > 7 glasses per week (Maastricht), following Dutch Health Council guidelines (Netherlands 2006) (lower threshold for women).

Smoking was classified as daily tobacco use in the past 12 months (Maastricht), tobacco abuse/dependence (Leuven and Maastricht) or positive smoking status (Maastricht).

### 2.4 Statistics

All analyses were conducted in R (v4.4.1). Data from all sites were combined to increase statistical power, with site‐specific distributions provided in Table S3.

Categorical variables (sex, parental education, alcohol use, smoking, cannabis use and stressful events) were compared using Pearson's chi‐square tests. Sleep quality, measured as a continuous, site‐standardised score, was compared both between sexes and across sites using the Wilcoxon rank‐sum test with continuity correction. IQ differences were assessed via the Mann–Whitney test due to non‐normality in VIQ and FSIQ. Spearman correlations examined associations between continuous variables, including IQ scores and environmental factors.

ANOVA assessed the effects of parental education, substance use, sleep patterns and stress on baseline IQ, adjusting for age at first assessment, sex and site. Multiple linear regression further explored significant predictors while controlling for the same covariates.

IQ scores from three visits per participant were analysed using linear mixed‐effects models with random intercepts to account for repeated measures within individuals. We included a fixed ‘timepoint’ term to test for overall mean changes in FSIQ, VIQ and PIQ across the cohort and then used estimated marginal means (EMMs) and post hoc pairwise comparisons to determine which timepoints differed significantly.

To capture any non‐linear trajectory, we also fitted a quadratic term (time^2^) in the same mixed‐effects framework.

Finally, we added demographic and environmental predictors and their interactions with time (while adjusting for age at assessment, sex and site) and used Type II ANOVA to determine which factors influence the overall IQ trajectory and, where interactions reached significance, computed EMMs with Bonferroni‐corrected pairwise contrasts to clarify how each predictor modified the IQ trajectory.

## 3 Results

### 3.1 Overview of Environmental Factors

Sample demographics and IQ scores of the total sample are displayed in Table 1.

**TABLE 1:** Overview of environmental factors within the sample across all three cohorts.

	*M* (SD)	Range	Missing (*N*)
Age	24 (14.0)	6–64	0
FSIQ (base)	73 (13.3)	36–109	60
VIQ (base)	75 (15.0)	47–114	129
PIQ (base)	75 (13.5)	45–119	128
Sleep score (z‐score)	0.006 (0.99)	−1.64 to 3.41	174

	Total sample, *N*	%	
Sex			0
Male	134	45.1	
Female	163	54.9	
Mother's education			77
High	62	28.2	
Middle	96	43.6	
Low	53	24.1	
No education	9	4.1	
Father's education			165
High	39	29.5	
Middle	61	46.2	
Low	31	23.5	
No education	1	0.8	
Alcohol use			75
Yes	10	4.5	
No	212	95.5	
Smoking			69
Yes	18	7.9	
No	210	92.1	
Cannabis use			158
Yes	3	2.2	
No	136	97.8	
Stressful events			175
Present	30	24.6	
Absent	92	75.4	

*Note:* It includes the mean (*M*) and standard deviation (SD) for age, sleep z‐scores and IQ measures, as well as the number of participants (*N*) or missing data (*N*) and percentage (%) for sex and various environmental measures.

We conducted a comprehensive overview of various environmental factors within the 22q11DS cohort, the results of which are detailed in Table 1. The majority of participants have mothers and fathers with middle or high educational levels, while only a small proportion have mothers (4.1%) or fathers (0.8%) with no formal education. Most participants abstain from alcohol (95.5%), smoking (92.1%), and cannabis use (97.8%). Additionally, there were no reports of other substance use in the cohorts. For 35%, worse‐than‐average sleep was registered, and 24.6% experienced traumatic events in their lives.

There were no significant sex differences in exposure to any of the environmental measures (Table S4).

### 3.2 Associations Between Different IQ Measures and Demographics

We examined the correlations between various IQ measures and explored the differences across sex and age groups.

Significant negative correlations were found between age and PIQ (*r*s = −0.15, *p* = 0.045) scores, indicating that as age increases, PIQ scores tend to decrease. Additionally, strong positive correlations were observed between FSIQ and PIQ (*r*s = 0.88, *p* < 0.001), FSIQ and VIQ (*r*s = 0.93, *p* < 0.001) and PIQ and VIQ (*r*s = 0.70, *p* < 0.001), which confirmed that these IQ measures are highly interrelated. No significant differences in IQ measures were identified between sex groups (*p* > 0.05).

### 3.3 Impact of Environmental Measures on Baseline IQ Scores

We performed a one‐way ANOVA on cross‐sectional, baseline IQ scores (FSIQ, PIQ and VIQ) to examine the impact of various environmental factors. The analysis identified significant effects of parental education on IQ scores. Specifically, lower levels of maternal education were significantly associated with lower VIQ (*p* = 0.02). Similarly, paternal education significantly influenced FSIQ (*p* = 0.004), VIQ (*p* < 0.001) and PIQ (*p* = 0.01).

**FIGURE 1:**
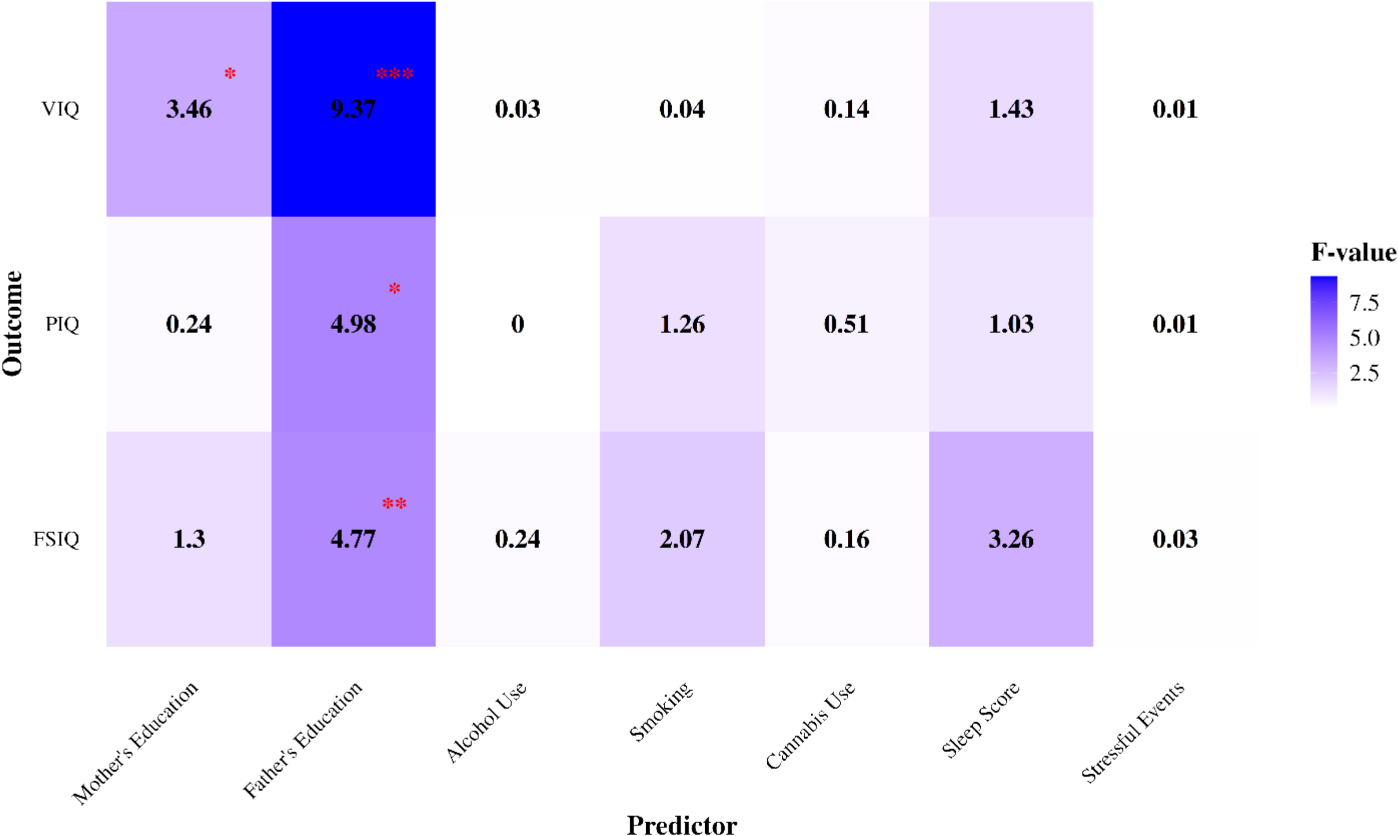
Results of ANOVA models assessing the effects of seven predictors on IQ scores (FSIQ, PIQ and VIQ). Each row represents one outcome variable (y‐axis), and each column represents a predictor (x‐axis). Colour intensity reflects the *F*‐value, with darker shades indicating stronger effects. Significant *p*‐values (*p* < 0.05) are displayed within the asterisks, highlighting predictors with significant associations. Covariates (age, site and sex) were included in the models but are not shown in the graph.

In contrast, other predictors, including alcohol use, smoking, cannabis use, sleep and stressful events, were found to have no statistically significant effect on IQ scores (Figure 1, Table S5).

Regression analyses revealed that parental education significantly influenced baseline IQ scores, with maternal and paternal education showing distinct effects. For maternal education, lower levels were associated with reduced VIQ, with significant effects observed for low (*p* = 0.003), middle (*p* = 0.039) and no education (*p* = 0.012). For paternal education, middle levels were significantly associated with reduced VIQ (*p* < 0.001) and FSIQ (*p* = 0.001) and low with FSIQ (*p* = 0.04) compared to high paternal education.

### 3.4 Changes in IQ Scores Over Time

**FIGURE 2:**
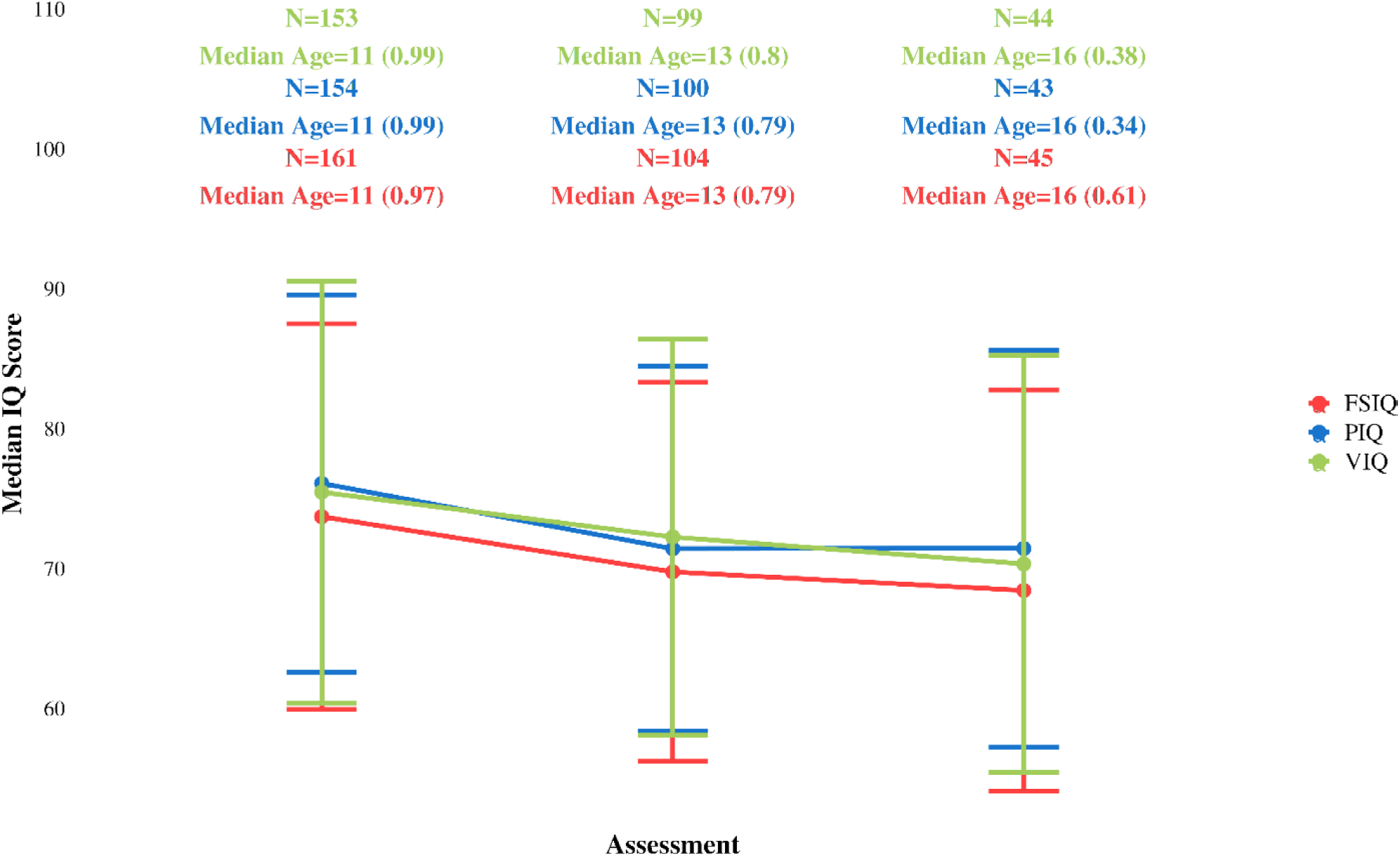
Full‐scale IQ (FSIQ), performance IQ (PIQ) and verbal IQ (VIQ) scores over three time points, with error bars representing variability. We reported the number of individuals (*N*) for each IQ score at a specific time point, along with the median age and standard error (SE) above the corresponding time point.

Changes in IQ scores over time were assessed using data from three visits per participant. The trajectory of these longitudinal IQ changes is documented in Figure 2 (along with the number of available data per timepoint) and in Table S6. The minimum interval between Timepoints 1 and 3 was 3 years, the maximum was 29 years and the mean interval was 7.87.

Linear mixed models revealed significant differences in FIQ scores across time points for all three measures (Time2: estimate = −3.34, *p* < 0.001; Time3: estimate = −6.17, *p* < 0.001). The PIQ also showed significant differences (Time2: estimate = −4.32, *p* < 0.001; Time3: estimate = −5.51, *p* < 0.001). Similarly, VIQ demonstrated significant changes (Time2: estimate = −2.94, *p* = 0.003; Time3: estimate = −6.85, *p* < 0.001).

Subsequent post hoc pairwise comparisons show significant differences between all specific pairs of time points, except for PIQ for Timepoints 2 and 3.

When we added the quadratic term, the linear contrast remained highly significant and negative for all IQ measures (FSIQ, PIQ and VIQ), confirming a steady decline over time, while the quadratic contrast was non‐significant in each model, indicating no detectable acceleration or deceleration in that decline. All models also showed substantial random‐intercept variance, reflecting notable individual differences in baseline IQ.

### 3.5 Impact of Demographic and Environmental Factors on IQ Scores Over Time

We addressed our main hypothesis in a longitudinal framework by investigating how environmental variables predicted within‐subject IQ change over time. The linear mixed model analysis with Type II ANOVA identified the following significant predictors for IQ measures: paternal education was significantly associated with reduced FSIQ, χ^2^(2) = 12.72, *p* = 0.002, VIQ, χ^2^(2) = 23.09, *p* < 0.001, and PIQ, χ^2^(2) = 7.02, *p* = 0.03. Maternal education also had significant effects on VIQ, χ^2^(3) = 13.08, *p* = 0.005, with lower levels of maternal education being linked to reduced verbal IQ.

The Type II ANOVA on the mixed‐effects model revealed a significant overall interaction between time and maternal education on VIQ, χ^2^(6) = 19.32, *p* = 0.004, indicating that the trajectory of verbal IQ over the three assessments differed by maternal education level. EMMs showed that at the first visit, participants whose mothers had a high education level had a mean VIQ of 85.8 (SE = 3.7) versus 71.0 (SE = 3.0) in the low‐education group (*t* = 3.37, *p* = 0.006). By the third visit, VIQ had declined to 78.5 (SE = 4.8) in the high‐education group and to 68.6 (SE = 4.2) in the low‐education group, although that pairwise difference was no longer significant after Bonferroni correction (*t* = 1.86, *p* = 0.39).

## 4 Discussion

### 4.1 Summary of Key Findings

This study examined environmental influences on IQ among 22q11DS carriers across European cohorts, using both cross‐sectional and longitudinal data. We hypothesised that higher levels of parental education, better sleep quality, fewer traumatic experiences, and lower substance use would be associated with improved IQ. Our cross‐sectional ANOVA analysis confirmed that lower levels of maternal education were associated with lower VIQ, and parental middle level of education was linked to lower VIQ and FSIQ. In contrast, sleep quality, substance use, and traumatic events had no significant association with IQ.

### 4.2 Parental Education and Gene–Environment Link

Our findings align with prior research highlighting the influence of parental education on cognitive outcomes in individuals with neurodevelopmental disorders (Reiss 2013; Peverill et al. 2020) and parallels general‐population studies showing parental education's impact on children's verbal IQ (Neiss and Rowe 2000). In 22q11DS families, however, parental education likely reflects both environmental support (cognitive stimulation, home resources) and inherited genetic factors.

Because maternal mental health may influence both parenting capacity and child IQ outcomes, some of the maternal‐education effect we observe could be mediated through caregiver stress rather than purely educational resources (Rindermann and Ceci 2018). Mothers of 22q11DS children face elevated mental health challenges (Niarchou et al. 2023), which may reduce capacity for promoting cognitive development. Systemic barriers, such as delays in obtaining special educational services, further disadvantage families with lower education. Moreover, parental education itself is heritable (Lee et al. 2018), as well as intelligence (Savage et al. 2018), and parents may harbour 22q11.2‐related risk alleles. Thus, our observed education‐IQ association probably represents a combination of direct environmental enrichment and gene–environment correlation.

Parental cognitive performance also plays a crucial role in shaping the cognitive abilities of offspring with 22q11.2DS, as evidenced by robust proband‐parent correlations (Fiksinski et al. 2022). Several genes within the 22q11.2 region encode proteins involved in key neurobiological processes affecting cognition. For instance, COMT variants influence dopamine metabolism and have been linked to working memory deficits (Gothelf et al. 2005; Armando et al. 2012). TBX1 (Gao et al. 2013; Hiramoto et al. 2022), PRODH (Carmel et al. 2014), HIRA (Jeanne et al. 2021), and MRPL40 (Devaraju et al. 2017) play crucial roles in biological processes associated with cognitive function and are implicated in the cognitive and educational challenges observed in individuals with 22q11.2DS. Environmental factors could interact with the phenotypical changes caused by the deletion of these genes, potentially exacerbating cognitive and educational difficulties. For example, parental education can enhance cognitive development by providing a stimulating home environment, fostering positive attitudes towards learning, and facilitating access to educational resources. This support can help mitigate some of the genetic challenges faced by individuals with 22q11DS, such as those associated with the COMT gene, which is linked to cognitive control and executive function deficits (Armando et al. 2012; Satterfield et al. 2018), and the PRODH gene, which is associated with impairments in working memory and cognitive flexibility (Li et al. 2008).

In our study, we observed that a significant proportion of parents of participants with 22q11DS had middle educational levels, with 43.6% of mothers and 46.2% of fathers falling into this category. In contrast, only 4.1% of mothers and 0.8% of fathers had no formal education. Previously, it was shown that socioeconomic status significantly influenced cognition in individuals with 22q11DS, with lower SES associated with poorer cognitive and behavioural outcomes (Shashi et al. 2010; Wolstencroft et al. 2022; Pimenta et al. 2024). This correlation is potentially owed to a reduced opportunity to access crucial educational, support, and healthcare resources necessary for cognitive development (Shashi et al. 2010) in the lower SES group. Since lower parental education levels may indicate socioeconomic disadvantages and barriers to accessing necessary support and/or interventions, such as specialised healthcare services, educational support, and therapeutic resources, this could introduce a recruitment bias in clinical cohorts of 22q11DS. This bias is pertinent to this study and may also be a common issue in clinical studies at large. Additionally, even though we used parental education as our SES variable, caregiving demands in 22q11DS may disrupt typical income and employment trajectories; therefore, parental education alone may not fully capture socioeconomic reality in this population.

### 4.3 Age‐Related IQ Trajectories and Comorbidities

Negative correlations between age and PIQ suggest age‐related declines in this cognitive domain, potentially reflecting accelerated cognitive ageing (Salthouse 2009). However, it is important to note that our analysis used standardised IQ scores, which are norm‐referenced to account for typical age‐related changes in cognitive performance. These findings may therefore reflect a growing‐into‐deficits phenomenon, where individuals with 22q11DS increasingly diverge from age‐matched norms as they age due to changes in normative data over time (Chawner et al. 2017). Genetic vulnerabilities of 22q11DS, coupled with health‐related factors, may drive this pattern.

Neurodegenerative processes, as evidenced by the association of 22q11.2DS with early‐onset Parkinson's disease (Boot et al. 2018) and shorter life expectancy (Bassett et al. 2009; Van et al. 2019), complicate the interpretation of age effects.

Importantly, we did not adjust for co‐occurring neurodevelopmental or psychiatric diagnoses, nor major medical comorbidities, all of which are prevalent in 22q11DS and strongly influence cognition. Consequently, some variance attributed to age or parental education may instead reflect unmeasured comorbidities. Future longitudinal studies should incorporate these clinical covariates to isolate true age‐related cognitive trajectories.

### 4.4 Trauma, Substance Use and Sleep

Although 24.6% of participants reported traumatic life events (consistent with elevated PTSD risk in 22q11DS; Martorell and Tsakanikos 2008), we found no significant trauma–IQ relationship. Trauma's cognitive impact may be mediated by unmeasured psychiatric comorbidities, which were not modelled here.

Substance use rates were very low (alcohol abstinence 95.5%; non‐smoking 92.1%; cannabis abstinence 97.8%). This likely reflects strong caregiver oversight, reduced peer influence (Vingerhoets et al. 2019), and COMT‐related genetic influences on risk‐taking (Bearden et al. 2005). Previous work suggests that carrying the 22q11.2 deletion itself, along with lower sensation‐seeking and altered reward processing, contributes to protective effects against substance use (Vingerhoets et al. 2019). Correspondingly, we observed no significant association between substance use and any IQ measure in this cohort.

About 35% of participants reported sleep disturbance. Despite well‐documented links between sleep and cognition (Arganbright et al. 2020; Moulding et al. 2020; Hyde et al. 2021; Chawner et al. 2023; O'Hora et al. 2023), we saw no direct sleep‐IQ association.

In 22q11DS, however, sleep disturbance often reflects underlying medical issues, such as congenital heart defects, palate anomalies, or neuropsychiatric medication, so does not represent directly an environmental factor (O'Hora et al. 2023). Consequently, any presumed link between sleep and IQ is likely mediated by these health conditions rather than representing an independent environmental effect.

### 4.5 Limitations and Strengths

The study has several limitations. A key limitation of our study is the lack of information on whether the 22q11.2 deletion was inherited or occurred *de novo*. This distinction is relevant, as previous research suggests that individuals with inherited deletions may exhibit different phenotypic trajectories compared to those with *de novo* deletions, potentially due to genetic background effects or familial environmental influences (Wolstencroft et al. 2022; Cillo et al. 2024). Without this information, our findings on environmental measures in 22q11DS may be confounded by underlying genetic factors associated with inheritance patterns. Our study is also limited by the lack of parental IQ data, which prevents us from distinguishing genetic influences from the environmental effects of parental education on cognitive outcomes in 22q11DS. Since some environmental measures are inherently linked to familial and genetic background, it remains challenging to fully disentangle true environmental effects from underlying inherited factors in our models. Another limitation of our study is the absence of a control group, which restricts our ability to compare the observed IQ directly with changes in 22q11DS individuals to a non‐affected population. Second, merging datasets from different cohorts introduces variability in questionnaire administration, resulting in data loss—a necessary consequence of analysing heterogeneous data. Consequently, certain variables (e.g., additional SES indicators) could not be retained due to inconsistent data availability across cohorts. Third, incomplete data across variables adversely affects statistical analyses, potentially limiting the robustness of the findings. Also, the inclusion of participants across various age groups introduces heterogeneity, which, while beneficial for developmental studies, complicates the interpretation of the results. These limitations emphasise the need for cautious interpretation and improvements in the data collection strategies for rare diseases.

There are also several notable strengths. First, we employed a longitudinal design, which allows the examination of changes and trajectories over time, providing insights that would not be possible with a cross‐sectional approach. Second, comprehensive data collection and the integration of data from multiple sources enabled an increased sample size, enhancing the study's statistical power; however, we were unable to exclude participants taking medication or with psychiatric diagnoses without further reducing our sample size. The relatively large cohort size for this field further strengthened the study. Moreover, the inclusion of various environmental measures, which are seldom examined in the context of 22q11DS, adds a unique dimension to the research, allowing for a more thorough investigation of the panel of environmental factors rather than focusing on one or two variables.

## 5 Conclusion

Parental education modestly predicted IQ within this 22q11DS cohort, suggesting that socioeconomic variables may contribute to cognitive variability alongside the primary genetic deletion. Although approximately one‐third of participants reported sleep disturbances, this factor was not significantly associated with IQ in our sample, as well as the number of traumatic events. These results underscore the importance of considering both genetic and environmental influences and their interaction when studying cognitive outcomes in 22q11DS. Future research using standardised measures and richer SES data (e.g., income, educational support) is needed to disentangle genetic effects from environmental contributions.

## 6 Conflicts of Interest

The authors declare no conflicts of interest.

## Supporting information


**Table S1:** Sleep symptomatology in the Cardiff cohort. The numbers represent the number of participants who answered positively to a question and the overall number of respondents, with the percentage in brackets (%).
**Table S2:** Sleep parameters in individuals with 22q11.2 deletion syndrome (22q11DS) from the Maastricht cohort. Sleep duration (in hours) was approximated by calculating the interval between self‐reported bedtime and morning wake time.
**Table S3:** Results of demographic and environmental characteristics across three sites: Cardiff, Leuven and Maastricht. The table includes counts (*N*) for sex, parents' education, alcohol use, smoking, cannabis use and stressful events. Sleep quality, as a continuous, site‐standardised score, is compared across sites with the Wilcoxon rank‐sum test.
**Table S4:** Results of Chi‐squared tests examining the association between sex and environmental factors with Chi‐statistics, degrees of freedom (df) and *p*‐values (*p*). Sleep quality, as a continuous, site‐standardised score, is compared across sites with the Wilcoxon rank‐sum test.
**Table S5:** Results of ANOVA models predicting full‐scale IQ (FSIQ), verbal IQ (VIQ) and performance IQ (PIQ) scores. The table includes degrees of freedom (df), a sum of squares (sum Sq), mean squares (mean Sq), *F*‐values and *p*‐values for each predictor. Predictors include sleep, parental education, stressful events and substance use, with covariates (age, sex and site). Each row represents the output for a specific predictor within a given model (for 21 models).
**Table S6:** Mean (M) scores and standard deviation (SD) scores of full‐scale IQ (FSIQ), verbal IQ (VIQ) and performance IQ (PIQ) in three time points (Timepoints 1, 2 and 3). *N* represents the number of participants in each consecutive time point for each measure in all three cohorts.

## Data Availability

The data that support the findings of this study are available from the corresponding author upon reasonable request.
