# Supplementary material for: Parental Education Predicts Longitudinal IQ Trajectories in 22q11.2 Deletion Syndrome: A Three‐Cohort European Study
Source: J Intellect Disabil Res. 2025 Aug 4;69(11):1304–14. doi: 10.1111/jir.70025 (PMC12576363; doi:10.1111/jir.70025)
Supplement: Supplementary file 1 — Table S1: Sleep symptomatology in the Cardiff cohort. The numbers represent the number of participants who answered positively to a question and the overall number of respondents, with the percentage in brackets (%). Table S2: Sleep parameters in individuals with 22q11.2 deletion syndrome (22q11DS) from the Maastricht cohort. Sleep duration (in hours) was approximated by calculating the interval between self‐reported bedtime and morning wake time. Table S3: Results of demographic and environmental characteristics across three sites: Cardiff, Leuven and Maastricht. The table includes counts (N) for sex, parents' education, alcohol use, smoking, cannabis use and stressful events. Sleep quality, as a continuous, site‐standardised score, is compared across sites with the Wilcoxon rank‐sum test. Table S4: Results of Chi‐squared tests examining the association between sex and environmental factors with Chi‐statistics, degrees of freedom (df) and p‐values (p). Sleep quality, as a continuous, site‐standardised score, is compared across sites with the Wilcoxon rank‐sum test. Table S5: Results of ANOVA models predicting full‐scale IQ (FSIQ), verbal IQ (VIQ) and performance IQ (PIQ) scores. The table includes degrees of freedom (df), a sum of squares (sum Sq), mean squares (mean Sq), F‐values and p‐values for each predictor. Predictors include sleep, parental education, stressful events and substance use, with covariates (age, sex and site). Each row represents the output for a specific predictor within a given model (for 21 models). Table S6: Mean (M) scores and standard deviation (SD) scores of full‐scale IQ (FSIQ), verbal IQ (VIQ) and performance IQ (PIQ) in three time points (Timepoints 1, 2 and 3). N represents the number of participants in each consecutive time point for each measure in all three cohorts. [file JIR-69-1304-s001.docx]

**Supplements**

**Table S1.** Sleep symptomatology in the Cardiff cohort. The numbers represent the number of participants who answered positively to a question and the overall number of respondents, with the percentage in brackets (%).

| **Trait** | **Cardiff cohort** |
| --- | --- |
| **Any sleep problem** | 46/83 (55.42%) |
| **Insomnia** |  |
| Absent | 68/83 (81.93%) |
| 1-2 hours | 9/83 (10.83%) |
| >=2 hours | 6/83 (7.23%) |
| **Hypersomnia** |  |
| Absent | 79/82 (96.34%) |
| At least 2 activities | 3/82 (3.66%) |
| **Restless sleep** |  |
| Absent | 55/83 (66.27%) |
| Present | 28/83 (33.73%) |
| **Unrested from sleep** |  |
| Absent | 65/83 (78.31%) |
| Present | 18/83 (21.69%) |
| **Tiredness** |  |
| Absent | 74/83 (89.16%) |
| At least half the time | 8/83 (9.64%) |
| Almost all the time | 1/83 (1.20%) |
| **Fatigability** |  |
| Absent | 79/83 (95.18%) |
| Increased | 4/83 (4.82%) |
| **Nightmares** |  |
| Absent | 71/83 (85.54%) |
| At least 3 occasions | 12/83 (14.46%) |
| **Night Terrors** |  |
| Absent | 79/83 (95.18%) |
| Present | 4/83 (4.82%) |
| **Somnambulism** |  |
| Absent | 75/83 (91.46%) |
| Present | 7/83 (8.54%) |

**Table S2.** Sleep parameters in individuals with 22q11.2 deletion syndrome (22q11DS) from the Maastricht cohort. Sleep duration (in hours) was approximated by calculating the interval between self-reported bedtime and morning wake time.

| **Characteristics** | **Median** | **Minimum** | **Maximum** |
| --- | --- | --- | --- |
| **Time of sleep** | 10.00-10.30 p.m | < 9.30 p.m. | > 01.00 a.m. |
| **Sleep onset latency** | 15-30 min | 0-5 min | 2-4 hours |
| **Number of night wakings** | 2 | 0 | >5 |
| **Length of night wakings** | 15-30min | 1-5min | 45min-1hour |
| **Morning wake time** | 06.30-07.00 hours | <05.00 hour | 10.30-11.00 hours |
| **Time between waking and getting out of bed** | 5-15min | 0-5min | 1-2 hours |
| **Number of hours of sleep** | 7-8 hours | 2-3 hours | >12 hours |
| **Subjective sleep quality** | 2 | -3 | 3 |

**Table S3.** Results of demographic and environmental characteristics across three sites: Cardiff, Leuven, and Maastricht. The table includes counts (N) for sex, parents' education, alcohol use, smoking, cannabis use, and stressful events. Sleep quality, as a continuous, site-standardised score, is compared across sites with the Wilcoxon rank‐sum test.

| **Category** | **Cardiff** | **Leuven** | **Maastricht** |
| --- | --- | --- | --- |
| **Sex** |  |  |  |
| Male | 50 | 23 | 61 |
| Female | 43 | 31 | 89 |
| **Mother's Education** |  |  |  |
| High | 23 | 11 | 28 |
| Middle | 31 | 33 | 32 |
| Low | 22 | 2 | 29 |
| No | 8 | 1 | 0 |
| **Father's Education** |  |  |  |
| High | 0 | 13 | 26 |
| Middle | 0 | 29 | 32 |
| Low | 0 | 0 | 31 |
| No | 0 | 0 | 1 |
| **Alcohol Use** |  |  |  |
| No | 76 | 54 | 82 |
| Yes | 0 | 0 | 10 |
| **Smoking** |  |  |  |
| No | 76 | 48 | 86 |
| Yes | 0 | 6 | 12 |
| **Cannabis Use** |  |  |  |
| No | 87 | 0 | 49 |
| Yes | 0 | 0 | 3 |
| **Sleep Score** | W-statistic = 1350, p = .651 | | |
| **Stressful Events** |  |  |  |
| Absent | 78 | 0 | 14 |
| Present | 4 | 0 | 26 |

**Table S4.** Results of Chi-squared tests examining the association between sex and environmental factors with Chi-statistics, degrees of freedom (df) and p-values (p). Sleep quality, as a continuous, site-standardised score, is compared across sites with the Wilcoxon rank‐sum test.

| **Environmental Factor** | **Chi statistics** | | **df** | **p-value** |
| --- | --- | --- | --- | --- |
| Site | 4.15 | 2 | | 0.13 |
| Mother's Education | 4.01 | 3 | | 0.26 |
| Father's Education | 1.70 | 3 | | 0.64 |
| Alcohol Use | 5.09E-30 | 1 | | 1.00 |
| Smoking | 0.40 | 1 | | 0.52 |
| Cannabis Use | 1.59E-04 | 1 | | 0.99 |
| Sleep Score | W-statistic = 1889, p =.994 | | | |
| Stressful Events | 1.87 | 1 | | 0.17 |

**Table S5.** Results of ANOVA models predicting full-scale IQ (FSIQ), verbal IQ (VIQ), and performance IQ (PIQ) scores. The table includes degrees of freedom (df), a sum of squares (sum Sq), mean squares (mean Sq), F-values, and p-values for each predictor. Predictors include sleep, parental education, stressful events, and substance use, with covariates (age, sex, and site). Each row represents the output for a specific predictor within a given model (for 21 models).

| **Output** | **Predictor** | **df** | **Sum.Sq** | **Mean. Sq** | **F.value** | **p -value** | **Model** |
| --- | --- | --- | --- | --- | --- | --- | --- |
| Mother’s Education | FSIQ | 3 | 578,39 | 192,80 | 1,30 | 0,28 | 1 |
| Age | FSIQ | 1 | 104,32 | 104,32 | 0,70 | 0,40 |  |
| Sex | FSIQ | 1 | 18,09 | 18,09 | 0,12 | 0,73 |  |
| Site | FSIQ | 2 | 1816,10 | 908,05 | 6,10 | 0,00 |  |
| Residuals | FSIQ | 191 | 28421,93 | 148,81 | NA | NA |  |
| Mother’s Education | VIQ | 3 | 2103,68 | 701,23 | 3,46 | 0,02 | 2 |
| Age | VIQ | 1 | 0,82 | 0,82 | 0,00 | 0,95 |  |
| Sex | VIQ | 1 | 10,94 | 10,94 | 0,05 | 0,82 |  |
| Site | VIQ | 2 | 768,48 | 384,24 | 1,90 | 0,15 |  |
| Residuals | VIQ | 126 | 25499,94 | 202,38 | NA | NA |  |
| Mother’s Education | PIQ | 3 | 102,15 | 34,05 | 0,24 | 0,87 | 3 |
| Age | PIQ | 1 | 58,59 | 58,59 | 0,42 | 0,52 |  |
| Sex | PIQ | 1 | 12,92 | 12,92 | 0,09 | 0,76 |  |
| Site | PIQ | 2 | 2152,22 | 1076,11 | 7,69 | 0,00 |  |
| Residuals | PIQ | 127 | 17781,79 | 140,01 | NA | NA |  |
| Father’s Education | FSIQ | 3 | 1817,13 | 605,71 | 4,77 | 0,00 | 4 |
| Age | FSIQ | 1 | 1037,22 | 1037,22 | 8,17 | 0,01 |  |
| Sex | FSIQ | 1 | 42,05 | 42,05 | 0,33 | 0,57 |  |
| Site | FSIQ | 1 | 68,02 | 68,02 | 0,54 | 0,47 |  |
| Residuals | FSIQ | 110 | 13962,78 | 126,93 | NA | NA |  |
| Father’s Education | VIQ | 2 | 3522,66 | 1761,33 | 9,37 | 0,00 | 5 |
| Age | VIQ | 1 | 14,43 | 14,43 | 0,08 | 0,78 |  |
| Sex | VIQ | 1 | 62,14 | 62,14 | 0,33 | 0,57 |  |
| Site | VIQ | 1 | 55,90 | 55,90 | 0,30 | 0,59 |  |
| Residuals | VIQ | 46 | 8649,56 | 188,03 | NA | NA |  |
| Father’s Education | PIQ | 2 | 1041,52 | 520,76 | 4,98 | 0,01 | 6 |
| Age | PIQ | 1 | 217,86 | 217,86 | 2,08 | 0,16 |  |
| Sex | PIQ | 1 | 94,75 | 94,75 | 0,91 | 0,35 |  |
| Site | PIQ | 1 | 15,82 | 15,82 | 0,15 | 0,70 |  |
| Residuals | PIQ | 46 | 4807,48 | 104,51 | NA | NA |  |
| Alcohol Use | FSIQ | 1 | 44,02 | 44,02 | 0,24 | 0,62 | 7 |
| Age | FSIQ | 1 | 329,04 | 329,04 | 1,83 | 0,18 |  |
| Sex | FSIQ | 1 | 122,53 | 122,53 | 0,68 | 0,41 |  |
| Site | FSIQ | 2 | 1504,15 | 752,07 | 4,18 | 0,02 |  |
| Residuals | FSIQ | 200 | 35987,17 | 179,94 | NA | NA |  |
| Alcohol Use | VIQ | 1 | 7,10 | 7,10 | 0,03 | 0,86 | 8 |
| Age | VIQ | 1 | 4,06 | 4,06 | 0,02 | 0,89 |  |
| Sex | VIQ | 1 | 52,52 | 52,52 | 0,23 | 0,63 |  |
| Site | VIQ | 2 | 500,88 | 250,44 | 1,09 | 0,34 |  |
| Residuals | VIQ | 151 | 34789,08 | 230,39 | NA | NA |  |
| Alcohol Use | PIQ | 1 | 0,00 | 0,00 | 0,00 | 1,00 | 9 |
| Age | PIQ | 1 | 56,31 | 56,31 | 0,33 | 0,57 |  |
| Sex | PIQ | 1 | 107,12 | 107,12 | 0,63 | 0,43 |  |
| Site | PIQ | 2 | 3610,91 | 1805,45 | 10,63 | 0,00 |  |
| Residuals | PIQ | 151 | 25637,56 | 169,79 | NA | NA |  |
| Smoking | FSIQ | 1 | 368,12 | 368,12 | 2,07 | 0,15 | 10 |
| Age | FSIQ | 1 | 268,53 | 268,53 | 1,51 | 0,22 |  |
| Sex | FSIQ | 1 | 99,84 | 99,84 | 0,56 | 0,45 |  |
| Site | FSIQ | 2 | 1326,48 | 663,24 | 3,73 | 0,03 |  |
| Residuals | FSIQ | 206 | 36643,58 | 177,88 | NA | NA |  |
| Smoking | VIQ | 1 | 9,90 | 9,90 | 0,04 | 0,84 | 11 |
| Age | VIQ | 1 | 3,22 | 3,22 | 0,01 | 0,91 |  |
| Sex | VIQ | 1 | 55,39 | 55,39 | 0,24 | 0,62 |  |
| Site | VIQ | 2 | 474,06 | 237,03 | 1,03 | 0,36 |  |
| Residuals | VIQ | 151 | 34811,08 | 230,54 | NA | NA |  |
| Smoking | PIQ | 1 | 213,22 | 213,22 | 1,26 | 0,26 | 12 |
| Age | PIQ | 1 | 49,82 | 49,82 | 0,29 | 0,59 |  |
| Sex | PIQ | 1 | 96,59 | 96,59 | 0,57 | 0,45 |  |
| Site | PIQ | 2 | 3400,62 | 1700,31 | 10,01 | 0,00 |  |
| Residuals | PIQ | 151 | 25651,64 | 169,88 | NA | NA |  |
| Cannabis Use | FSIQ | 1 | 30,46 | 30,46 | 0,16 | 0,69 | 13 |
| Age | FSIQ | 1 | 40,91 | 40,91 | 0,21 | 0,65 |  |
| Sex | FSIQ | 1 | 26,98 | 26,98 | 0,14 | 0,71 |  |
| Site | FSIQ | 1 | 358,19 | 358,19 | 1,84 | 0,18 |  |
| Residuals | FSIQ | 128 | 24900,46 | 194,53 | NA | NA |  |
| Cannabis Use | VIQ | 1 | 32,97 | 32,97 | 0,14 | 0,71 | 14 |
| Age | VIQ | 1 | 152,72 | 152,72 | 0,66 | 0,42 |  |
| Sex | VIQ | 1 | 100,93 | 100,93 | 0,44 | 0,51 |  |
| Site | VIQ | 1 | 34,74 | 34,74 | 0,15 | 0,70 |  |
| Residuals | VIQ | 113 | 25964,06 | 229,77 | NA | NA |  |
| Cannabis Use | PIQ | 1 | 97,18 | 97,18 | 0,51 | 0,48 | 15 |
| Age | PIQ | 1 | 0,60 | 0,60 | 0,00 | 0,96 |  |
| Sex | PIQ | 1 | 79,82 | 79,82 | 0,42 | 0,52 |  |
| Site | PIQ | 1 | 591,48 | 591,48 | 3,12 | 0,08 |  |
| Residuals | PIQ | 114 | 21625,75 | 189,70 | NA | NA |  |
| Sleep Score | FSIQ | 1 | 546.61 | 546.61 | 3.26 | 0.07 | 16 |
| Age | FSIQ | 1 | 45.68 | 45.68 | 0.27 | 0.60 |  |
| Sex | FSIQ | 1 | 82.09 | 82.09 | 0.49 | 0.49 |  |
| Site | FSIQ | 1 | 88.84 | 88.84 | 0.53 | 0.47 |  |
| Residuals | FSIQ | 111 | 18616.92 | 167.72 | NA | NA |  |
| Sleep Score | VIQ | 1 | 318.77 | 318.77 | 1.43 | 0.24 | 17 |
| Age | VIQ | 1 | 6.21 | 6.21 | 0.03 | 0.87 |  |
| Sex | VIQ | 1 | 63.31 | 63.31 | 0.28 | 0.60 |  |
| Site | VIQ | 1 | 664.48 | 664.48 | 2.97 | 0.09 |  |
| Residuals | VIQ | 84 | 18774.76 | 223.51 | NA | NA |  |
| Sleep Score | PIQ | 1 | 180.86 | 180.86 | 1.03 | 0.31 | 18 |
| Age | PIQ | 1 | 156.44 | 156.44 | 0.89 | 0.35 |  |
| Sex | PIQ | 1 | 0.01 | 0.01 | 0.00 | 1.00 |  |
| Site | PIQ | 1 | 263.31 | 263.31 | 1.50 | 0.22 |  |
| Residuals | PIQ | 85 | 14915.44 | 175.48 | NA | NA |  |
| Stressful Events | FSIQ | 1 | 4,01 | 4,01 | 0,03 | 0,87 | 19 |
| Age | FSIQ | 1 | 226,91 | 226,91 | 1,49 | 0,22 |  |
| Sex | FSIQ | 1 | 86,87 | 86,87 | 0,57 | 0,45 |  |
| Site | FSIQ | 1 | 0,17 | 0,17 | 0,00 | 0,97 |  |
| Residuals | FSIQ | 114 | 17361,73 | 152,30 | NA | NA |  |
| Stressful Events | VIQ | 1 | 1,75 | 1,75 | 0,01 | 0,92 | 20 |
| Age | VIQ | 1 | 133,29 | 133,29 | 0,69 | 0,41 |  |
| Sex | VIQ | 1 | 38,46 | 38,46 | 0,20 | 0,66 |  |
| Site | VIQ | 1 | 2172,27 | 2172,27 | 11,20 | 0,00 |  |
| Residuals | VIQ | 78 | 15126,09 | 193,92 | NA | NA |  |
| Stressful Events | PIQ | 1 | 0,88 | 0,88 | 0,01 | 0,94 | 21 |
| Age | PIQ | 1 | 129,32 | 129,32 | 0,96 | 0,33 |  |
| Sex | PIQ | 1 | 10,78 | 10,78 | 0,08 | 0,78 |  |
| Site | PIQ | 1 | 2243,59 | 2243,59 | 16,59 | 0,00 |  |
| Residuals | PIQ | 79 | 10684,99 | 135,25 | NA | NA |  |

**Table S6.** Mean (M) scores and standard deviation (SD) scores of full-scale IQ (FSIQ), verbal IQ (VIQ), and performance IQ (PIQ) in three time points (Timepoint 1,2 and 3). N represent the number of participants in each consecutive time point for each measure in all three cohorts.

|  | **Timepoint 1** | **Timepoint 2** | **Timepoint 3** |
| --- | --- | --- | --- |
| **FSIQ** (N=161, 104, 45) | 73.7 (13.8) | 69.8 (13.5) | 68.4 (14.3) |
| **VIQ** (N =153, 99, 44) | 75.5 (15.1) | 72.3 (14.2) | 70.3 (14.9) |
| **PIQ** (N=154, 100, 43) | 76.1 (13.5) | 71.4 (13.0) | 71.4 (14.2) |
